# Hints for Hot Weather

**Published:** 1891-06

**Authors:** 


### HALL'S JOURNAL OF HEALTH

TRUTH DEMANDS NO SACRIFICE; ERROR CAN MAKE NONE.

Vol. 38. JUNE, 1891. No. 6.

### HINTS FOR HOT WEATHER.

Comfort in summer depends almost as much on the food and drink that are taken into the system as on the clothing. If a man is careful about his diet, in particular about what he drinks, he may be reasonably comfortable in summer, though he sits around all day with a silk hat, a tight fitting collar, and frock coat. The unpleasant sensations of a hot day come as well from inside the body as outside. The feeling of parching thirst in the throat is more uncomfortable than the direct rays of the sun. The feeling that a man has eaten something that does not agree with him, or has had too many cold drinks, causes greater discomfort than torrents of perspiration. To be comfortable, there must be an equilibrium between the interior apparatus of the body and the external conditions and circumstances. No man who eats and drinks carelessly on a hot day will have this comfortable poise.

A mistake many men make is not to drink until they are thirsty, and not to drink any thing at all cool until they are hot. A man may get up in the morning and feel fairly comfortable ; he will eat his breakfast and drink but little. When he gets to his work it will be warmer and he will be warmer ; he will then drink beer, soda water, lemonade, or ice water. In a little while he will be thirstier and he will drink some more of the same. From this time on, as the thermometer rises, he will become hotter and thirstier. It is not that he has not taken enough fluid to quench any amount of thirst, but that the fluid was not taken until he was thirsty, and therefore does not quench his thirst at once. Eating satisfies hunger almost at once, but the food is not taken into the system for several hours, until the digestion and assimilation are completed. It is so with thirst even more than with hunger. Thirst is a local feeling, but it means that there is not enough water in the system. Pouring water down the throat puts water into the stomach, but not into the system for some time afterward, particularly if the water is cold. Coffee and tea quench thirst more rapidly when they are hot than when they are cold, because they are assimilated more quickly.

Then after the victim of the heat is loaded up with cold water he begins to perspire more freely. This means that he has put into himself more water than his system demands, that the heat and his overloaded system combined are working it off. As the perspiration trickles down his face, he goes and takes more drinks, and so keeps the uncomfortable round until night, when he tosses around damp and uncomfortable.

In the same way that a short run exhausts a man's breath and makes him sweat more than a long leisurely walk, so a number of iced drinks poured in rapidly tire a man more, and make him sweat more, than a moderate amount of fluids taken at long intervals of time. The best time to drink is in the morning. The stomach is empty, and the fluids will be carried through the system and into the blood more speedily. A pint of moderately cool water then will do more to relieve thirst during the day than ten glasses of beer or soda water in the afternoon. A man is somewhat like a locomotive; if the water is not judiciously administered to the boiler, there is an explosion. The time to lay in the supply of fluids is before the man starts for the day. If he wants to drink any more, he may drink it slowly, and should not have it so cold that it will inflame the throat and the stomach, and make them warmer. A cold bath makes the skin glow with warmth. So a cold bath of ice water, in its reaction, warms the throat and stomach.

